# Synthesis of Poly(l-lactide-co-ε-caprolactone) Copolymer: Structure, Toughness, and Elasticity

**DOI:** 10.3390/polym13081270

**Published:** 2021-04-14

**Authors:** Mengyuan Zhang, Zhonghua Chang, Xiaofeng Wang, Qian Li

**Affiliations:** 1 School of Materials Science and Engineering, Zhengzhou University, Zhengzhou 450002, China; artemis8827@163.com; 2 National Center for International Research of Micro-Nano Molding Technology, Key Laboratory of Henan Province for Micro Molding Technology, Zhengzhou 450002, China; 13253660150@163.com; 3 School of Mechanics Science and Safety Engineering, Zhengzhou University, Zhengzhou 450002, China

**Keywords:** poly(l-lactide-co-ε-caprolactone), synthesis, segment, toughness, elasticity

## Abstract

Biodegradable and bioabsorbable polymers have drawn considerable attention because of their mechanical properties that mimic human soft tissue. Poly(l-lactide-co-ε-caprolactone) (PLCL), the copolymer of L-lactic (LA) and ε-caprolactone (CL), has been applied in many tissue engineering and regenerative medicine fields. However, both the synthesis of PLCL and the structure-activity relationship of the copolymer need to be further investigated to allow tuning of different mechanical properties. The synthesis conditions of PLCL were optimized to increase the yield and improve the copolymer properties. The synthetic process was evaluated by while varying the molar ratio of the monomers and polymerization time. The mechanical properties of the copolymer were investigated from the macroscopic and microscopic perspectives. Changes in the polymerization time and feed ratio resulted in the difference in the LA and CL content, which, in turn, caused the PLCL to exhibit different properties. The PLCL obtained with a feed ratio of 1:1 (LA:CL) and a polymerization time of 30 h has the best toughness and elasticity. The developed PLCL may have applications in dynamic mechanical environment, such as vascular tissue engineering.

## 1. Introduction

Biodegradable polyester materials have gained increasing attention for their potential applications in the biomedical field [1]. Among them, polyester materials synthesized from ring-opening polymerization (ROP) of cyclic ester monomers [2] are widely used in surgical sutures [3], drug delivery carriers [4], bone fixation [5], and tissue engineering scaffolds [6].

Poly(lactic acid) (PLA) and poly(ε-caprolactone) (PCL) polyester materials have been extensively studied [7,8]. The advantages of their biodegradability, non-toxic degradation products, good biocompatibility, and drug permeability, have attracted widespread attention [9,10]. However, PLA exhibits high strength and poor toughness, while PCL exhibits good toughness and low strength [11,12,13]. Poly(l-lactide-co-ε-caprolactone) (PLCL) can not only combine the advantages of both but also improve strength and toughness. 

Many medical devices are implanted in the dynamic mechanical environment of the human body. They need to adapt to states of continuous relaxation and contraction to maintain their own performance without mechanical stimulation of the surrounding tissues [14,15]. The elastic properties of PLCL can meet this requirement [16].

The reactivity of the two monomers is very different in the ring opening copolymerization (ROP) process of poly(l-lactide-co-ε-caprolactone) [17,18,19,20]. Therefore, the synthesis conditions have a great influence on the molecular characteristics of poly(l-lactide-co-ε-caprolactone). Grijpma et al. [21] found that the change of polymerization temperature could affect the average sequence length (lactic (LA) segment and caprolactone (CL) segment) of the PLCL copolymer. Fernandez et al. [22] researched the influence of the polymerization temperature and feed ratio on PLCL copolymers and found that changing the polymerization temperature and feed ratio affected the LA content of PLCL copolymers, which, in turn, affected the average sequence length, average relative molecular weight, tensile properties, and aging behavior of PLCL copolymer. At the same time, they researched the influence of the amount of catalyst, polymerization temperature, and polymerization time on the average sequence length and degree of randomness of PLCL copolymers, and the effect of different catalyst types on the cytological behavior of PLCL copolymers are also considered [23]. Contreras et al. [24] researched the use of zinc diphenyl as initiator to prepare PLCL block copolymers using sequential polymerization, and changed the ratio of comonomers to make them have a low dispersibility and a good ordering. Mezzasalma et al. [25] found that using benzoic acid as a catalyst and various alcohols as initiators, PLCL copolymers with good molar mass and dispersibility can be prepared under solvent-free conditions. The elongation at breaking point of the synthesized PLCL copolymer could only reach to 485% [22,26].

In this work, several statistical poly(l-lactide-co-ε-caprolactone) copolymers at a polymerization temperature of 150 °C were synthesized by ROP. SnOct_2_ was added as catalyst, and the initiator was not used under different polymerization conditions, that is, varying the polymerization times and the feed molar ratios. The aim is to optimize the synthesis process and mechanical properties of poly(l-lactide-co-ε-caprolactone) by evaluating the polymerization time and feed molar ratio to obtain PLCL with high toughness, good elasticity and high yield. We synthesize a series of PLCL copolymers and compare them with a commercial PLCL copolymer. ^1^HNMR, FT-IR, gel permeation chromatography (GPC), and differential scanning calorimetry (DSC) are applied to characterize the molecular structure of the copolymers. Tensile, cyclic tensile, and rheological tests, along with dynamic thermomechanical analyzer (DMA) and atomic force microscope (AFM), were applied to investigate the mechanical properties of the copolymers.

## 2. Experimental

### 2.1. Materials

The L-lactide (L-LA) monomer was supplied by Yikeside Technology Co., Ltd. (Tianjin, China). The ε-caprolactone (ε-CL) monomer (assay ≥ 99%) and Stannous octoate (assay ≥ 95%) were both provided by Aladdin Biochemical Technology Co., Ltd. (Shanghai, China). The commercial poly(l-lactide-co-ε-caprolactone) sample (LA/CL ratio 50:50, intrinsic viscosity of 3.9 dL/g) was purchased from Daigang Biomaterials Co., Ltd. (Jinan, China). Ethyl acetate, ethanol and dichloromethane were obtained from Fengchuan Chemical Reagent Technology Co., Ltd. (Tianjn, China)

### 2.2. Synthesis of PLCL

The PLCL copolymer was synthesized by an ROP. Ethyl acetate was used to purify L-LA beforehand. In each polymerization, predetermined amounts of the purified L-LA and ε-CL were simultaneously added and melted in the reaction flask. The flask was purged for a period of time with a nitrogen stream under the surface of the melt. Then, the stannous octoate catalyst was added using a 3000:1 co-monomers: catalyst molar ratio. The flask was placed in an oil bath, and the reaction was carried out with continuous stirring. After the reaction, the reactant was dissolved in dichloromethane and precipitated with excess ethanol. Finally, the precipitate was dried in a vacuum oven at 40 °C until it reached constant weight.

### 2.3. Preparation of PLCL Films

Films of the PLCL copolymers were prepared by solution casting. About 1.5 g of PLCL was dissolved in 15 mL of methylene chloride to make a solution, placed in a petri dish, and dried for 48 h at room temperature to obtain a PLCL film.

### 2.4. Characterization of PLCL

#### 2.4.1. ^1^HNMR

A 600 MHz nuclear magnetic resonance spectrometer (AVIII HD 600, Bruker, Karlsruhe, Baden-Wurttemberg, Germany) was used to obtain the ^1^HNMR spectrum of the PLCL copolymer with deuterated chloroform (CDCl_3_) as the solvent and tetramethylsilane as the internal standard.

#### 2.4.2. FT-IR

An infrared spectrometer (NEXUS 470, Nicolet Instrument Company, Madison, WI, USA) was used to acquire the FT-IR spectrum of the PLCL copolymer. The sample powder was ground and pressed with potassium bromide before the spectrum was recorded. The scanned range was 4000 to 400 cm^−1^, the resolution was 4 cm^−1^, and the number of repeat scans was 10.

#### 2.4.3. GPC

A Waters 1414 chromatograph apparatus was used to obtain the gel permeation chromatography (GPC) measurements of the PLCL copolymers. The Styragel columns were calibrated with polystyrene standards at 50 mV of signal. Tetrahydrofuran was used as the mobile phase at a flow rate of 1.0 mL/min at room temperature. Number average molecular weights (Mn), weight average molecular weights (Mw), and polydispersity index (Ð) data of the synthesized PLCL copolymers were obtained from their molecular weight distribution curve at a RI peak height in the range of 40 to 60 mV.

#### 2.4.4. DSC

A differential scanning calorimetry (DSC) tester (Q200, TA Company, Newcastle, DE, USA) was used to obtain DSC data for the PLCL copolymer. The weight of a single test sample was about 5 mg. In a nitrogen environment, the sample was heated from −70 °C to 180 °C at a temperature increase rate of 10 °C/min, then cooled to −70 °C at a temperature decrease rate of 10 °C/min, and, finally, heated again, at a temperature rise rate of 10 °C/min, to 180 °C.

#### 2.4.5. Tensile Test

A universal tensile testing machine (UTM2203, Shenzhen Suns Technology Co., Ltd., Shenzhen, China) was used to perform tensile tests and cyclic tensile tests on the PLCL films at room temperature. The inlet force was set to 0.01 N, the stretching rate to 40 mm/min, and the number of cycles to 20. The test as repeated at least five times for each group of samples.

The reduction of the elastic properties for PLCL copolymers was calculated as follows:

(1) $$\frac{\mathrm{Rr}\left(1\right)-\mathrm{Rr}\left(N\right)}{\mathrm{Rr}\left(1\right)}\;\times \;100\%.$$

#### 2.4.6. Rheological Test

A rotational rheometer (TA DHR-2, TA Company, Newcastle, DE, USA) was used for testing the viscoelasticity of PLCL copolymers with a fixed gap of 1 mm between the 25 mm diameter parallel plates. In order to prevent thermal oxidative degradation, a frequency sweep was performed at 140 °C and 180 °C under nitrogen flow. The angular frequency sweep range was 100 to 0.01 rad/s, and the fixed strain was 1%.

#### 2.4.7. DMA

A dynamic thermomechanical analyzer (Q800, TA Company, Newcastle, DE, USA) was used to carry out dynamic mechanical measurements in tensile mode. The PLCL samples were heated from −60 to 100 °C at a rate of 3 °C/min. A frequency of 10 Hz was used. The displacement and force amplitude were maintained at 20 µm and 0.3 N, respectively.

#### 2.4.8. AFM

An atomic force microscope (Keysight 7500, Agilent, Palo Alto, CA, USA) was used, in the tapping mode, to capture the surface topography of the PLCL films. The phase diagram was obtained to show the difference of a local position of the PLCL films.

## 3. Results and Discussion

### 3.1. Synthesis of PLCL

**Figure 1:**
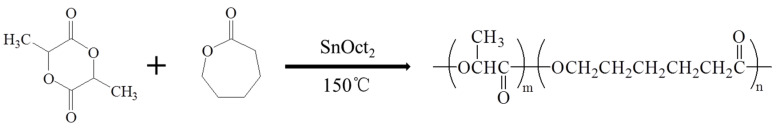
The synthesis procedure of poly(l-lactide-co-ε-caprolactone) (PLCL) copolymer.

Given its good activity, catalytic effect, and FDA (Food and Drug Administration, Silver Spring, MD, USA) permission, SnOct_2_ was selected to catalyze the ROP synthesis of L-LA and ε-CL to PLCL [27]. The CL section has very low reactivity below 140 °C, such that it is not easy to open the ring [28]. Hence, a polymerization temperature of 150 °C was chosen. The reaction process is shown in Figure 1. The monomer: catalyst molar ratio of 3000:1 (based on a serial of preliminary experiment) was fixed in order to research the effects of different reaction times and feed molar ratios on the synthesis.

**Figure 2:**
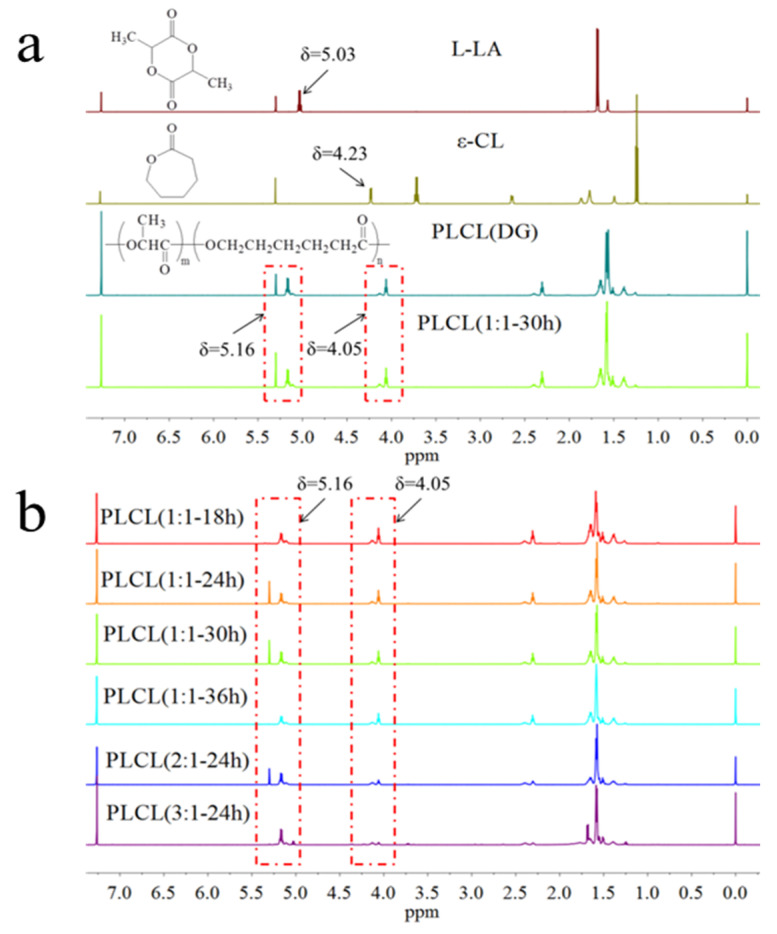
^1^HNMR spectra of L-lactide (L-LA), ε-caprolactone (ε-CL), PLCL (DG), and PLCL (1:1-30 h) (**a**); ^1^HNMR spectra of PLCL copolymers synthesized at different conditions (**b**).

The ^1^HNMR spectra of L-LA and ε-CL are shown in Figure 2a. In the ^1^HNMR spectrum of L-LA, the quadruple splitting peak with δ = 5.03 ppm comes from the –CH group [29]. In the ^1^HNMR spectrum of ε-CL, the triple splitting peak of δ = 4.23 ppm comes from the –CH_2_ connected to the ester group.^23^ The ^1^HNMR spectra of the commercial PLCL (denoted DG) and the synthesized PLCL (1:1-30 h) are shown in Figure 2a. The –CH peak for LA was observed at δ = 5.16 ppm and the –CH_2_ peak in –CL at δ = 4.05 ppm, indicating that L-LA and ε-CL underwent the ROP successfully. The integrals of the two peak areas were calculated, revealing the composition of the PLCL copolymer. ^1^HNMR spectra of the PLCL synthesized under different conditions are shown in Figure 2b. Calculating the composition molar ratio of the synthesized PLCL as the reaction time increased from 18 h to 36 h, it showed that the relative content of LA increased. A maximum was reached when the reaction time was 30 h, after which the content decreased. The greater was the feed molar ratio of LA to CL, the greater was the content of LA in the PLCL (Table 1). When the feed molar ratio of LA:CL was 3:1, the content of LA in PLCL (3:1**-**24 h) was at its maximum.

**Figure 3:**
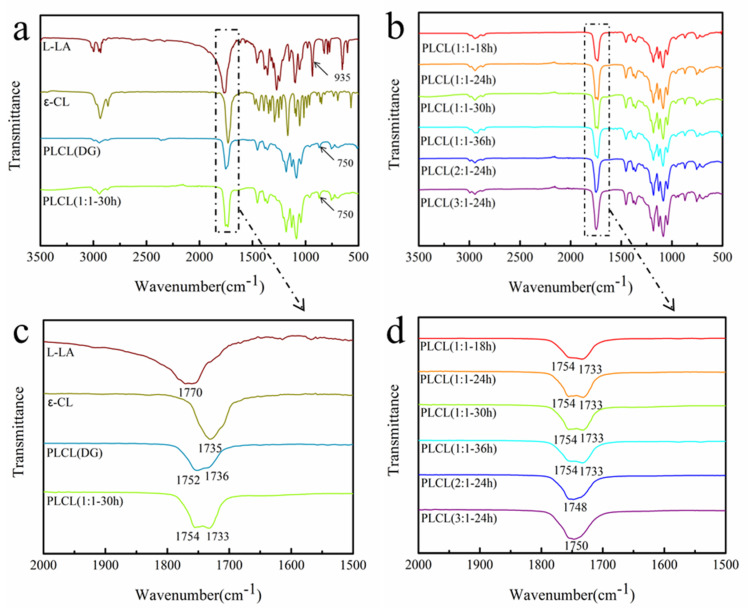
FT-IR spectra of L-LA, ε-CL, PLCL (DG) and PLCL (1:1-30 h) (**a**); FT-IR spectra of PLCL copolymers synthesized under different conditions (**b**); FT-IR spectra of L-LA, ε-CL, PLCL (DG) and PLCL (1:1-30 h) in the region of 1500 to 2000 cm^−1^ (**c**); FT-IR spectra of PLCL copolymers synthesized under different conditions in the region of 1500 to 2000 cm^−1^ (**d**).

The FT-IR spectra of L-LA and ε-CL are shown in Figure 3a. In the spectrum of L-LA, the characteristic band of C=O was observed at about 1770 cm^−1^, and the characteristic band of the –C–H from the ring skeleton was at about 935 cm^−1^ [30]. In the spectrum of ε-CL, the band at about 1735 cm^−1^ was the characteristic band of C=O [31]. The FT-IR spectra of the commercial PLCL and the synthesized PLCL (1:1**-**30 h) are shown in Figure 3a. The PLCL did not show the characteristic band of –CH from the L-LA ring skeleton at 934 cm^−1^, indicating that the ROP reaction of L-LA had occurred. The existence of the characteristic band of –CH_2_ at 750 cm^−1^ demonstrated that there were CL structural units present. The two absorption peaks at about 1754 cm^−1^ and 1733 cm^−1^ were assigned to the C=O in both LA and CL (Figure 3c). In other words, the ROP of L-LA and ε–CL was carried out and the PLCL copolymers were obtained successfully. The FT-IR spectra of PLCL copolymers synthesized under different conditions are shown in Figure 3b. As the reaction time increased over the range of 18 h to 36 h, the characteristic band of C=O shifted to higher wave numbers initially, and then to lower wave numbers (Figure 3d). This was because the wave number of the characteristic band of C=O was affected by the LA content. The higher the LA content, the higher the wave number of the characteristic band of C=O. Therefore, the characteristic band of C=O also shifted to a higher wave number with the increase of the feed molar ratio (LA:CL).

**Figure 4:**
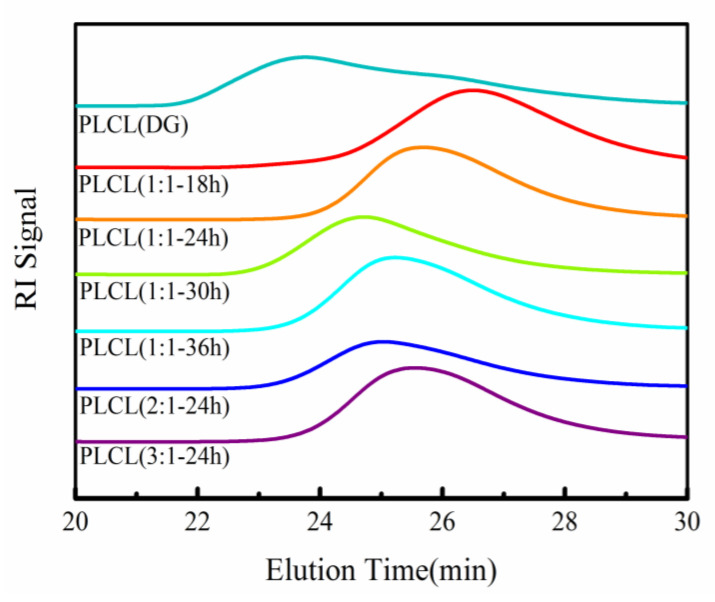
Gel permeation chromatography (GPC) curves of PLCL copolymers.

The GPC curves of the PLCL copolymers are given in Figure 4. There was a single peak on each GPC curve, suggesting the successful ring-opening copolymerization of L-LA and ε-CL. The average molecular weight of the PLCL copolymer varied with the reaction time. As the reaction time was increased, the average molecular weight of the PLCL copolymer increased initially and then decreased (Table 1). The reaction time had a greater impact on the reaction-the reaction could be incomplete if the reaction time was too short. Thus, the PLCL copolymer had its minimum average molecular weight (Mn = 20.33 × 10^3^ g/mol) and lowest yield (76.44%) for the shortest polymerization time (18 h). Some products may depolymerize when the reaction time is too long. Hence, the number average molecular weight of the PLCL copolymer was 37.97 × 10^3^ g/mol after the longest polymerization time (36 h). Among the PLCL copolymers synthesized under different conditions, PLCL (1:1**-**30 h) possessed the maximum average molecular weight (Mn = 53.81 × 10^3^ g/mol) and highest yield (87.34%, Table 1).

**Figure 5:**
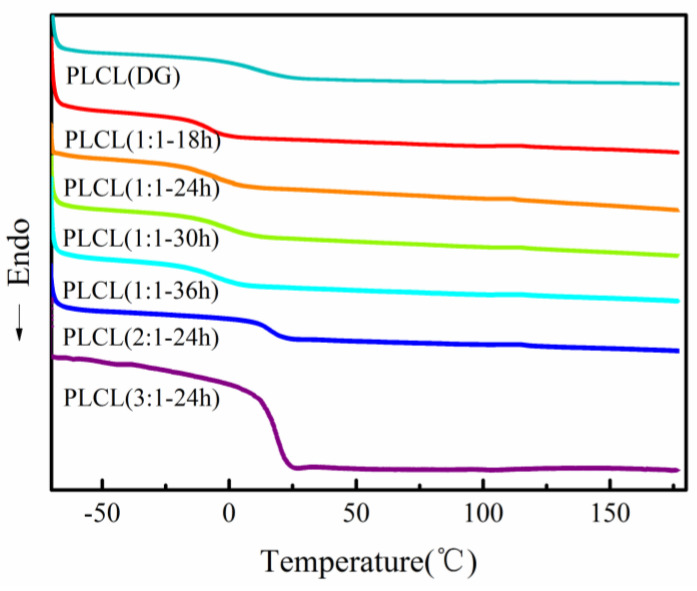
Differential scanning calorimetry (DSC) of PLCL copolymers.

DSC was chosen to confirm the aggregate structure of PLCL copolymers. As shown in Figure 5, there was no melting peak in the PLCL copolymers. This indicated that the PLCL copolymer was amorphous. This was due to the transesterification reaction during the polymerization of the LA and CL segments, which led to the copolymerization. Neither of the co-monomers could produce chain segments regular enough to fold together regularly, preventing any crystallinity [32]. The T_g_ of PLA was 57.6 °C [33], and the T_g_ of PCL was −62 °C [34]. The T_g_ of the PLCL copolymer firstly increased and then decreased as the reaction time was increased from 18 to 36 h. PLCL (1:1-18 h) had the lowest T_g_ (−12.77 °C), and PLCL (1:1**-**30 h) the highest T_g_ (1.98 °C), when varying only the reaction time. The T_g_ of the PLCL copolymer also increased with the increase of the LA:CL feed molar ratio. PLCL (3:1**-**24 h) had the highest T_g_ (18.51 °C) when varying the feed molar ratio. The T_g_ of the PLCL copolymer was greatly influenced by the LA content. As the LA content increased, the T_g_ of the PLCL copolymer also increased due to the higher rigidity of the LA segments.

### 3.2. Mechanical Properties

Ideally, a complete description of the elastic properties of any material should include its measured resilience and elongation at break [15,35]. And then, the resilience can be expressed by the strain recovery rate. In other words, the higher the strain recovery rate of a material, the higher the elongation at break, and the better its elasticity. Moreover, it should have the enough mechanical strength to meet application requirements. To understand the mechanical properties of the PLCL copolymers, a universal tensile testing machine was used to test PLCL samples prepared by solution casting. PLCL (1:1**-**18 h) was very sticky and could not be made into a film by solution casting, so it could not be tested with the universal tensile testing machine.

**Figure 6:**
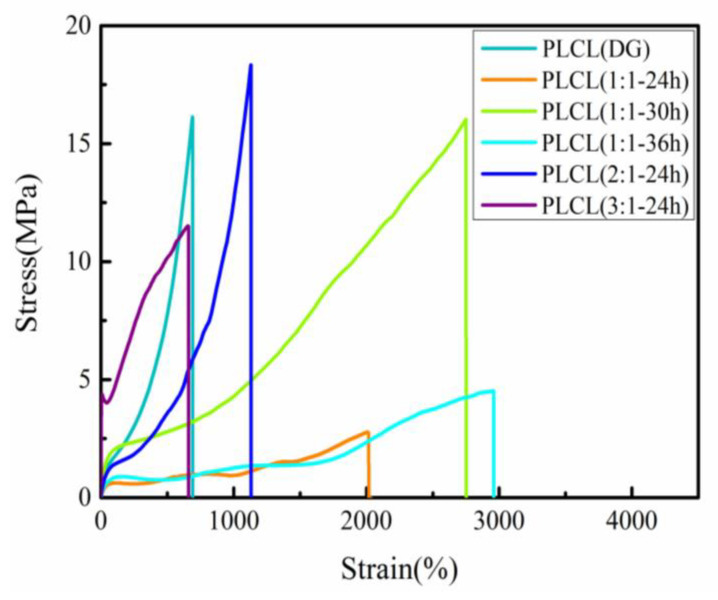
Stress–strain curves of the films of PLCL copolymers.

PLCL copolymers with different molar ratios and reaction times demonstrated dramatically different mechanical properties (Figure 6). Table 2 gives the corresponding data calculated from the curves, i.e., the elastic moduli, tensile strengths, and elongation at the breaking point. With increasing reaction time (from 24 h to 36 h), the elastic moduli and tensile strengths increased initially and then decreased. For the elongation at the breaking point, there was almost no difference between the different PLCL copolymers. PLCL (1:1**-**30 h) had the highest elastic modulus (8.5 ± 0.6 MPa) and tensile strength (15.1 ± 0.9 MPa). This was because the content of the LA content was increased in the PLCL chains with the increase of the reaction time. The average molecular weight of copolymer was closely related to its mechanical properties [28]. Thus, the higher average molecular weight of PLCL (1:1**-**36 h) than PLCL (1:1**-**24 h) led to its higher elastic modulus and tensile strength. The LA content increased with the feed molar ratio of LA to CL. Hence, the elastic modulus of the PLCL copolymer gradually increased and the elongation at the breaking point gradually reduced. PLCL (3:1**-**24 h) had the highest elastic modulus (46.2 ± 5.9 MPa) and the lowest elongation at the breaking point (705.1 ± 70.7%) when varying the feed molar ratio. On the other hand, the Mn and Mw of PLCL (3:1**-**24 h) were lower than PLCL (2:1**-**24 h), giving it a lower tensile strength. Of all the synthesized PLCL copolymers, PLCL (1:1-30 h) had the highest LA content and the highest average molecular mass (Mn = 53.81 × 10^3^ g/mol), and hence showed the most desirable mechanical properties. These results suggest that the composition of PLCL can be altered by varying the conditions during the synthesis process, resulting in changes in the mechanical properties. In addition, compared with commercial PLCL (DG), PLCL (1:1**-**30 h) had both a lower elastic modulus and higher elongation at the breaking point. Although the tensile properties of the two polymer samples were almost the same, PLCL (1:1**-**30 h) exhibited the better softness and toughness (Figure S1).

**Figure 7:**
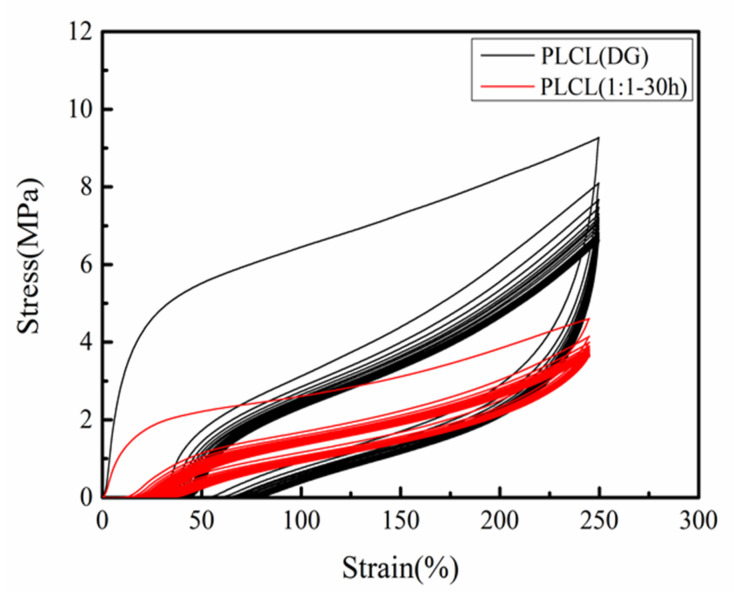
Cyclic stretch curves of PLCL (DG) and PLCL (1:1-30 h) samples.

PLCL copolymers have been extensively applied in the tissue engineering filed, within cyclic mechanical environments, such as vascular tissue engineering scaffolds [15]. To further explore the elastic properties of PLCL (1:1**-**30 h) and PLCL (DG), cyclic tensile tests were performed. The cyclic stretching curve of PLCL (DG) and PLCL (1:1**-**30 h), with a strain of 250%, are shown in Figure 7. In the cyclic stretching process, the strain could not recover to the original state at the end of each cycle. As the cycle number increased, the resultant irreversible deformation went up. This was due to the stress softening effect of PLCL films [36]. The strain recovery rate [37] at 250% strain, was used to characterize the elastic properties (Table 3). Rr (N) gives the strain recovery rate at 250% strain for the Nth cycle. As the number of cycles increased, the strain recovery rate of the materials gradually declined. As shown in Table 3, the maximum strain recovery rate of PLCL (1:1**-**30 h) was 93.5%, and the minimum strain recovery rate was 88.3%; and the maximum strain recovery rate of PLCL (DG) was 79.8%, and the minimum strain recovery rate was 69.9%. According to Equation (1), to calculate the reduction of the elastic properties for PLCL copolymers, we could see that the elastic properties had a reduction of 12.4% after 20 stretch cycles for PLCL (DG), whereas the reduction was only 5.7% for PLCL (1:1**-**30 h). Besides, the stretch-recovery processes of PLCL (DG) and PLCL (1:1**-**30 h) was shown in Videos S1 and S2. The results suggested that the elastic properties of PLCL (1:1**-**30 h) were better than PLCL (DG). This could make it more favorable in applications, such as vascular tissue engineering.

### 3.3. Thermodynamic Properties

**Figure 8:**
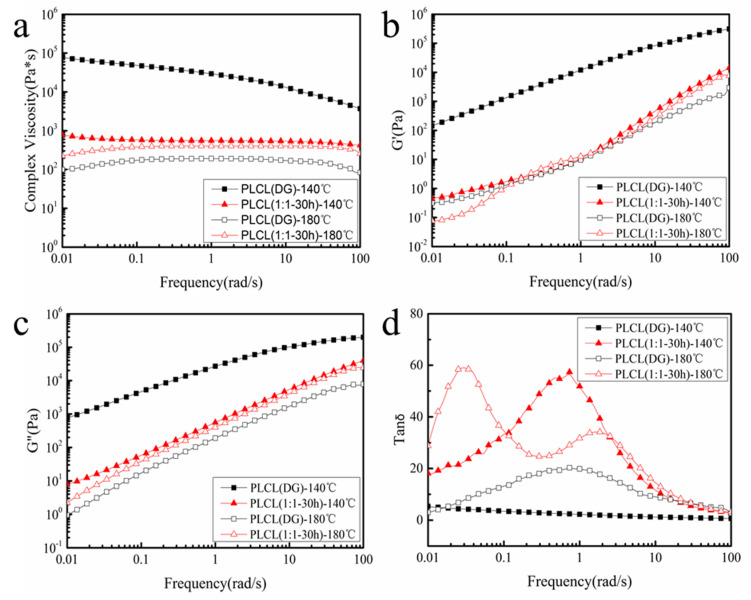
Complex viscosity (**a**), storage modulus (**b**), loss modulus (**c**), and tan δ (**d**) of neat PLCL (DG) and PLCL (1:1-30 h) samples obtained by dynamic frequency sweep at 140 °C and 180 °C.

Rheological tests were carried out to investigate the viscoelasticity of PLCL (DG) and PLCL (1:1**-**30 h). Figure 8 shows the results from a rotational rheometer frequency sweep at 140 °C and 180 °C, respectively. As shown in Figure 8a, the complex viscosity of PLCL (DG) was higher than that of PLCL (1:1**-**30 h), and its shear thinning tendency was more obvious in the high frequency region at 140 °C. This was due to PLCL (DG) having the higher weight average molecular weight and polydispersity. Figure 8b,c compare the G’ and G” of PLCL (DG) and PLCL (1:1**-**30 h) at 140 °C and 180 °C. PLCL (DG) gave higher G’ and G” curves than PLCL (1:1**-**30 h) at 140 °C. We attribute this increase in the moduli to the lesser LA content in PLCL (1:1**-**30 h). In other words, PLCL (1:1**-**30 h) contained more CL segments, which contributed to its flexibility. However, at 180 °C, the viscoelastic melt behavior was much more pronounced in PLCL (1:1**-**30 h). PLCL (1:1**-**30 h) gave slightly higher G’ and G” curves than PLCL (DG) at this temperature. Furthermore, the storage modulus of PLCL (1:1**-**30 h) in the low frequency region was lower than PLCL (DG), but it was higher than PLCL (DG) in the high frequency region. This might be due to the micro-phase separation of some LA and CL segments in PLCL (1:1**-**30 h) at 180 °C (Figure 8d).

**Figure 9:**
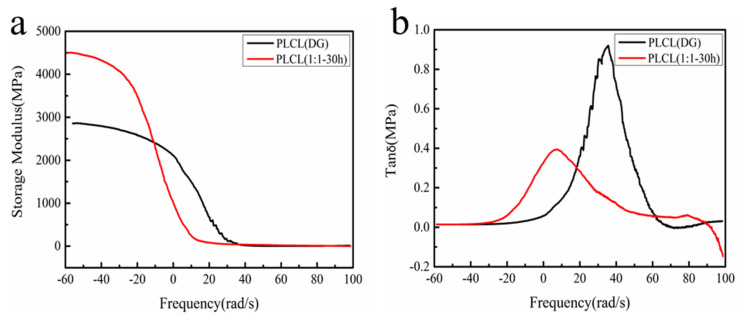
Temperature dependence of the dynamic mechanical spectra for the storage modulus (**a**) and tan δ (**b**) for PLCL (DG) and PLCL (1:1-30 h).

Figure 9 shows the temperature dependence of the dynamic mechanical spectra of PLCL (DG) and PLCL (1:1**-**30 h) obtained by DMA measurement of the storage modulus and tan δ. For PLCL (DG) and PLCL (1:1**-**30 h), a single drop and a single peak are found in the storage modulus and tan δ curves, respectively. This was due to the glass transition for both PLCL (DG) and PLCL (1:1**-**30 h) [5]. Moreover, the storage modulus of PLCL (1:1**-**30 h) was higher than that of PLCL (DG), indicating that the elasticity of PLCL (1:1-30 h) was better. The peak value of tan δ for PLCL (1:1-30 h) was lower than that of PLCL (DG), suggesting that PLCL (1:1**-**30 h) possessed a lower glass transition temperature and, thus, a better elasticity. This was due to the higher CL content in PLCL (1:1**-**30 h) than in PLCL (DG). The DMA results agreed well with the cyclic tensile test in that PLCL (1:1**-**30 h) showed a lower damping effect than that of PLCL (DG). The elasticity of PLCL (1:1**-**30 h) demonstrates that it is a desirable biodegradable elastomer for tissue engineering and regenerative medicine.

### 3.4. Surface Morphology

**Figure 10:**
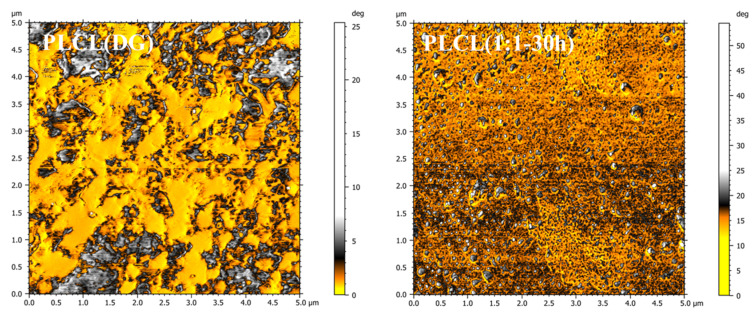
Phase images of PLCL (DG) and PLCL (1:1-30 h) across an area of 5 μm × 5 μm.

AFM was used to obtain phase information on the material surfaces via the force interaction between the probe and the material surface [38]. As seen in Figure 10, there were many blocky, gray-white phase regions in the PLCL (DG) phase diagram, caused by the phase separation of the LA and CL segments. However, the phase image of PLCL (1:1**-**30 h) showed a relatively uniform color throughout the scanned area, suggesting a single-component phase. We attribute the holes in the PLCL (1:1**-**30 h) phase diagram to the hole defects caused by solvent volatilization when making the PLCL films. Thus, the phase distribution of PLCL (1:1**-**30 h) was more uniform than that of PLCL (DG). This might be one of the reasons why the mechanical properties of PLCL (1:1**-**30 h) were better than those of PLCL (DG). At the same time, this also explained why the contact angle of PLCL (1:1**-**30 h) was slightly lower than that of PLCL (DG) (Figure S2).

## 4. Conclusions

An ROP method was used to synthesize PLCL copolymers. We explored the influence of changes in synthesis process conditions on the structure and properties of the synthesized products by changing the parameters of reaction time and feed ratio. The results show that the synthesis process conditions have a great influence on the molecular structure of PLCL copolymers. When the process conditions were monomer: catalyst molar ratio of 3000:1, reaction time of 30 h, and feed molar ratio of 1:1(LA:CL), the synthesized PLCL copolymer had the best mechanical properties and highest yield (>85%). Furthermore, compared a commercial PLCL copolymer, the synthesized PLCL (1:1**-**30 h) had better toughness and elasticity. As a biodegradable and bioabsorbable elastomer, PLCL has shown great promise for applications in tissue engineering, regenerative medicine, and other fields. This work gives insight into processes for tuning the toughness and elasticity of the copolymer to suit these applications.

## Figures and Tables

**Table 1:** Characterization of PLCL (DG) and PLCL copolymers synthesized at different conditions.

Sample	Feed Molar Ratio	Copolymer Composit Molar Ratio	Yield	Mn (×10^3^)	Mw (×10^3^)	Ð	Tg
	LA:CL	LA:CL	%	g/mol	g/mol		°C
PLCL (DG)	1:1	3.18:1	/	54.46	231.2	4.25	9.14
PLCL (1:1-18 h)	1:1	2.18:1	76.44	20.33	43.79	2.15	−12.77
PLCL (1:1-24 h)	1:1	2.28:1	88.78	29.85	57.81	1.94	−4.39
PLCL (1:1-30 h)	1:1	2.46:1	87.34	53.81	120.0	2.23	1.98
PLCL (1:1-36 h)	1:1	2.22:1	82.75	37.97	78.85	2.08	−11.03
PLCL (2:1-24 h)	2:1	5.44:1	92.16	41.67	90.80	2.18	11.07
PLCL (3:1-24 h)	3:1	9.90:1	88.57	33.88	67.36	1.99	18.51

**Table 2:** Tensile properties of the films of PLCL copolymers.

Sample	Elastic Modulus (MPa)	Tensile Strength (MPa)	Elongation at the Breaking Point (%)
PLCL(DG)	12.0 ± 1.0	16.1 ± 3.2	689.5 ± 21.2
PLCL(1:1-18 h)	/	/	/
PLCL(1:1-24 h)	1.3 ± 0.3	2.7 ± 0.2	2611.7 ± 572.4
PLCL(1:1-30 h)	8.5 ± 0.6	15.1 ± 0.9	2661.3 ± 575.9
PLCL(1:1-36 h)	2.1 ± 0.4	3.8 ± 1.0	2860.0 ± 135.8
PLCL(2:1-24 h)	2.5 ± 0.6	18.9 ± 2.9	1131.7 ± 175.1
PLCL(3:1-24 h)	46.2 ± 5.9	12.1 ± 0.8	705.1 ± 70.7

**Table 3:** Elastic properties of PLCL (DG) and PLCL (1:1-30 h).

Sample	Rr(1) (%)	Rr(5) (%)	Rr(10) (%)	Rr(15) (%)	Rr(20) (%)
PLCL(DG)	79.8	74.4	72.5	71.1	69.9
PLCL(1:1-30 h)	93.5	90.7	89.5	88.9	88.3

## Supplementary Materials

The following are available online at https://www.mdpi.com/2073-4360/13/8/1270/s1, Figure S1: Tensile process of PLCL (DG) and PLCL (1:1-30 h). Figure S2: Water contact angle of PLCL (DG) and PLCL (1:1-30 h). Video S1: Stretch-recovery processes of PLCL (DG). Video S2: Stretch-recovery processes of PLCL (1:1-30 h).
