# Supplementary material for: Synthesis of Poly(l-lactide-co-ε-caprolactone) Copolymer: Structure, Toughness, and Elasticity
Source: Polymers (Basel). 2021 Apr 14;13(8):1270. doi: 10.3390/polym13081270 (PMC8070679; doi:10.3390/polym13081270)
Supplement: Supplementary file 1 [file polymers-13-01270-s001.zip › polymers-1167497-supplementary/polymers-1167497-supplementary.pdf]

polymers

Supporting Information

Synthesis of Poly(l-lactide-co-ε-caprolactone) Copolymer: Structure, Toughness, and Elasticity

Mengyuan Zhang [1,2], Zhonghua Chang [2,3], Xiaofeng Wang [2,3,*] and Qian Li [1,2,3,*]

1 School of Materials Science and Engineering, Zhengzhou University, Zhengzhou 450002, China; artemis8827@163.com (M.Z.)
2 National Center for International Research of Micro-Nano Molding Technology, Key Laboratory of Henan Province for Micro Molding Technology, Zhengzhou 450002, China; 13253660150@163.com (Z.C.)
3 School of Mechanics Science and Safety Engineering, Zhengzhou University, Zhengzhou 450002, China
* Correspondence: xiaofengwang@zzu.edu.cn (X.W.); qianli@zzu.edu.cn (Q.L.)

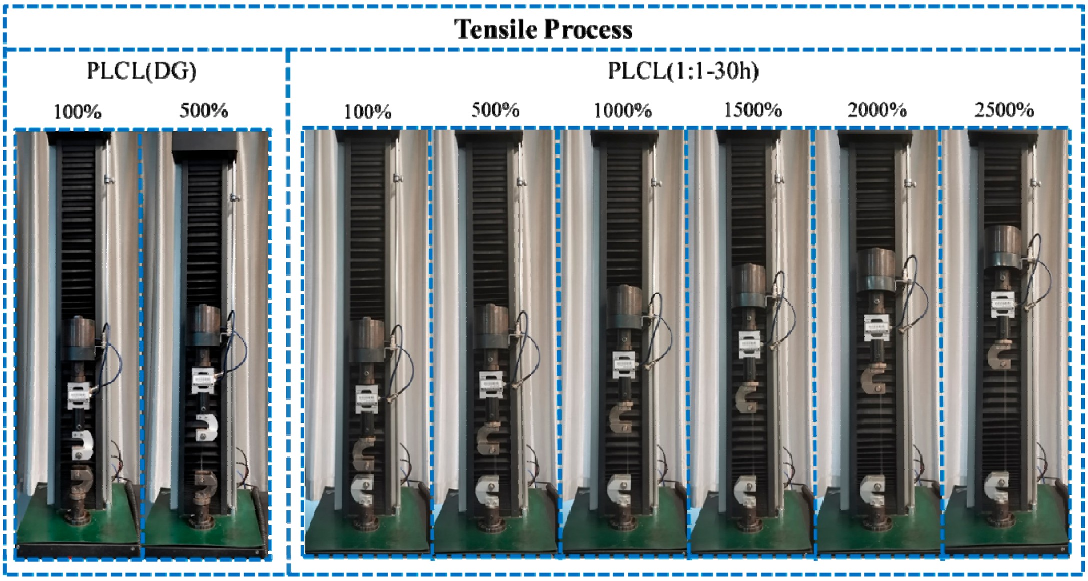

Figure S1. Tensile process of PLCL (DG) and PLCL (1:1-30h).

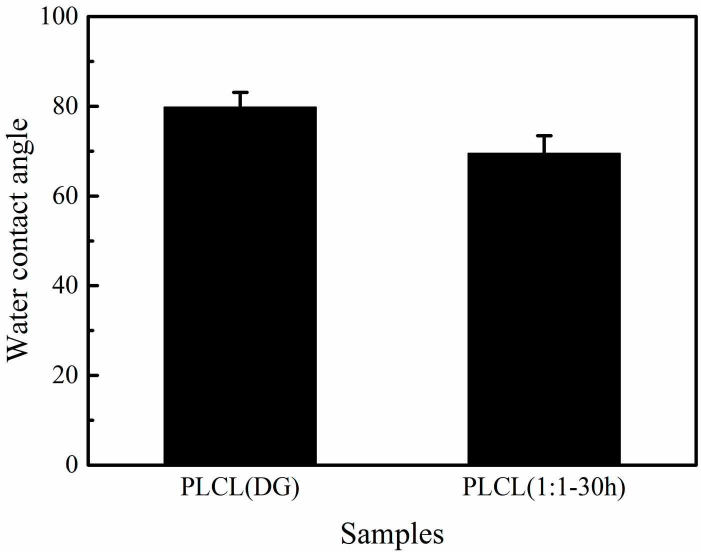

Figure S2. Water contact angle of PLCL (DG) and PLCL (1:1-30h).

PLCL(DG).mp4

Video S1. Stretch-recovery process of PLCL (DG).

PLCL(HM).mp4

Video S2. Stretch-recovery process of PLCL (1:1-30h).
